# Fibroblast growth factor-23 remodels vascular extracellular matrix via glycosaminoglycan induction: implications for calcification in chronic kidney disease

**DOI:** 10.1080/0886022X.2025.2567528

**Published:** 2025-10-06

**Authors:** Christian Freise, Tia Jernej, Susanne Metzkow, Jörg Schnorr, Matthias Taupitz

**Affiliations:** Department of Radiology, Charité – Universitätsmedizin Berlin, Berlin, Germany

**Keywords:** Chronic kidney disease, cardiovascular disease, extracellular matrix, fibroblast growth factor 23, glycosaminoglycans, vascular calcification

## Abstract

Chronic kidney disease (CKD) leads to accumulation of uremic toxins, which contribute to cardiovascular disease (CVD) and mortality. Among these, fibroblast growth factor 23 (FGF-23), a bone-derived hormone, is associated with arterial stiffness, vascular calcification, and left ventricular hypertrophy. However, the mechanisms linking elevated FGF-23 levels to vascular alterations remain poorly understood. We hypothesized that FGF-23 modulates the expression of sulfated glycosaminoglycans (sGAGs) and hyaluronic acid (HA) in vascular cells. Rat vascular smooth muscle cells (VSMCs) and human endothelial cells (ECs) were treated with FGF-23 ± its co-receptor Klotho and analyzed using qPCR, Western blotting, Blyscan assay, Alcian blue staining, ELISA, and reporter assays. FGF-23 significantly increased sGAG (2.5-fold) and HA (1.6-fold) levels in VSMCs, and sGAG (50-fold) and HA (3.7-fold) levels in ECs. Klotho alone induced a ∼72-fold rise in sGAGs in ECs but had no effect in VSMCs. FGF-23 also upregulated GAG-specific gene expressions of carbohydrate sulfotransferase 1 and xylosyltransferase 2 ∼1.6-fold and increased HA-specific hyaluronan synthase-2 and -3 protein expression. These effects were mediated by ERK and NF-κB signaling. To evaluate biological relevance, we assessed calcium- and phosphate-induced calcification in VSMCs. FGF-23 significantly enhanced calcification by ∼65%, which paralleled elevated sGAG levels. Inhibition of GAG sulfation with NaClO_3_ significantly reduced sGAGs and prevented FGF-23-induced calcification. Similarly, the FGFR inhibitor AZD4547 abolished FGF-23-induced increases in sGAGs and calcification in both VSMCs and ECs. These findings indicate that FGF-23 modulates vascular GAG composition and promotes calcification, thereby contributing to pathological vascular remodeling in CKD.

## 1. Introduction

Chronic kidney disease (CKD) is a progressive and irreversible condition defined by impaired kidney function and/or structure causing a declining glomerular filtration rate [1]. Patients with CKD have a significantly higher risk of cardiovascular disease (CVD), hospitalization for coronary heart disease, heart failure, ischemic stroke, and peripheral arterial disease as well as CVD-related mortality when compared to the general population [2]. During CKD, increased blood levels of uremic toxins (UTs), such as inorganic phosphate, urea and indoxyl phosphate are observed [3,4]. This results in cardiovascular damage, e.g., by inducing endothelial dysfunction, vascular wall calcification and loss of vasomotricity [3,5], increased susceptibility to infection, and neurologic manifestations [3,4]. Recently, glycosaminoglycans (GAGs) have been identified as indirect targets of UTs in vascular smooth muscle cells (VSMCs), endothelial cells (ECs), and extracellular vesicles [6,7].

GAGs are polysaccharide compounds attached to membrane bound core proteins or extracellular matrix proteins and function as cell signaling mediators, by interacting with signaling molecules and stabilizing ligand/receptor complexes [8]. This function stems from their negative charge achieved by carboxylic groups or different degrees of sulfation, which attracts positively charged molecules [8]. Changes in these properties of GAGs can affect the function and health of the cardiovascular system [9] and makes them interesting targets also for molecular imaging, such as by positively charged iron oxide nanoparticles [7,10]. Moreover, mice studies have shown that GAG overproduction in the aorta contributes to osteoblastic transformation of VSMCs, leading to vascular calcification, a hallmark of atherosclerosis and CVD [11]. By interacting with chemokines, GAGs are also linked to inflammation in general [12,13].

Besides established UTs, fibroblast growth factor 23 (FGF-23) has been discussed as another UT [14]. FGF-23 is a bone-secreted hormone, released in response to high serum levels of phosphate [5,15]. In physiological conditions, FGF-23 binds to the fibroblast growth factor receptor (FGFR) 1 in the kidneys, where the interaction is stabilized by the co-factor Klotho [5,15]. This allows activation of the Ras/mitogen-activated protein kinase (MAPK) pathway and downregulation of the sodium-phosphate co-transporter, resulting in lower phosphate reabsorption in the kidney [5,15]. However, impaired kidney function leads to chronically and progressively elevated levels of FGF-23, due to the kidney’s inability to excrete phosphate [5]. High FGF-23 levels have been associated with arterial stiffness, muscle calcification, cardiac fibrosis, and remodeling as well as left ventricular hypertrophy [16,17]. FGF-23 also plays a role in immune responses and is associated with inflammatory conditions, particularly in CKD patients [18,19]. Further, inflammation can stimulate FGF-23 gene transcription, leading to increased levels in the circulation [18]. In this context, it is noteworthy that systemic and vascular inflammation is a key factor for the progression of CVD and kidney disease in CKD. However, the specific mechanisms that link CKD with inflammation are still not fully understood and leave FGF-23 out of consideration for now [20].

Aiming to better understand the intermediate step between high FGF-23 levels and their effects on pathophysiological alterations in the cardiovascular system, we here evaluated whether FGF-23, similarly to other UTs [6,7], influences the sulfation of GAGs in VSMCs and ECs.

## 2. Materials and methods

### 2.1. Cell culture

Human ECs (EA.Hy926, ATCC^®^ CRL-2922™, Manassas, VA) and rat aortic VSMCs (A7r5, ATCC^®^ CRL-1444™, Manassas, VA) were cultured in standard culture medium which consisted of DMEM (Gibco/ThermoFisher Scientific, Hennigsdorf, Germany) with 1.0 g/L glucose, 50 μg/mL streptomycin, 50 units/mL penicillin, 1.8 mM calcium, 1.0 mM phosphate, 0.8 mM magnesium, and 10% heat-inactivated fetal bovine serum (FBS, Gibco, Hennigsdorf, Germany).

### 2.2. Treatment of cells with FGF-23

Cells were treated for up to seven days in multiwell plates (NUNC, Roskilde, Denmark) with standard culture medium supplemented with recombinant human FGF-23 (Abcam, Cambridge, UK) and/or Klotho (Sigma-Aldrich, Taufkirchen, Germany) as indicated in the figures. The medium was replaced every 2nd day. Cells treated with vehicles only served as control. A suitable concentration of FGF-23 for the treatment of EC and VMSC was derived from proliferation measurements using the CellTiter 96^®^ Non-Radioactive Cell Proliferation Assay (Promega, Walldorf, Germany) according to the manufacturer’s instructions.

### 2.3. Alcian blue staining of sulfated GAGs (sGAGs)

Cells in 12-well plates (NUNC, Roskilde, Denmark) were treated for five days without or with FGF-23 in standard culture medium. Then, the cells were washed with PBS and fixed with Roti-Histofix (Carl Roth, Karlsruhe, Germany) for 15 min. After the cells were washed two times with distilled water, they were stained with Alcian blue solution (pH 2.4; 700 μL per well) for 5 min. To wash the excess stain off, the cells were washed with 1 mL of distilled water for 2 min while gently mixing, at least three times or until the stain stopped dissociating from the cells. The cells were evaluated under light microscopy at 20× magnification. Alcian blue staining was quantified using Image J software (version 1.50i; National Institutes of Health, Bethesda, MA).

### 2.4. Measurement of sGAG levels in cells

Levels of sGAGs in cultured cells after 5 d were determined using the Blyscan Sulfated Glycosaminoglycan Assay (Biocolor, Belfast, UK) as described earlier [7].

### 2.5. Measurement of NF-κB activation in ECs and VSMCs

NF-κB activity in both cell lines was determined using a luciferase reporter assay as described before [21].

### 2.6. Analysis of HA levels in cell lysates by ELISA measurements

Levels of hyaluronic acid (HA) in cells were determined using the HA Quantikine™ ELISA Kit (R&D Systems, Minneapolis, MN) as described before [6].

### 2.7. Analysis of gene and protein expressions in VSMCs

Treatment-dependent gene and protein expressions in the cells were determined by quantitative real time PCR (Taqman) and Western blot, respectively. Table 1 lists all used Taqman probes and Table 2 lists the antibodies used for Western blot.

**Table 1.** List of applied Taqman-probes (ThermoFisher, Hennigsdorf, Germany) in the gene expression experiments.

Gene	Full name	Assay-ID (rat)	Assay-ID (human)
CHST1	Carbohydrate sulfotransferase 1	Rn01484520_m1	Hs04972213_s1
CHSY1	Chondroitin sulfate synthase 1	Rn01478125_m1	Hs00208704_m1
EXT1	Exostosin glycosyltransferase 1	Rn00468764_m1	Hs00609162_m1
HEXA	Hexosaminidase subunit alpha	Rn01422539_m1	Hs00942655_m1
RPL19	Ribosomal protein L19	Rn00821265_g1	Hs02338565_gH
SULF2	Sulfatase 2	Rn01423347_m1	Hs01016480_m1
XYLT2	Xylosyltransferase 2	Rn00574186_m1	Hs01048792_m1

**Table 2.** List of applied primary antibodies in the Western blot experiments.

Target	Dilution	Company	Product number
AKT	1:1,000	Cell Signaling (Danvers, MA)	#9272
p-AKT	1:1,000	Cell Signaling (Danvers, MA)	#4056
ERK	1:1,000	Cell Signaling (Danvers, MA)	#9102S
p-ERK	1:1,000	Cell Signaling (Danvers, MA)	#4370S
EXT1	1:1,000	ThermoFisher (Hennigsdorf, Germany)	#PA5-106907
GAPDH	1:2,000	ThermoFisher (Hennigsdorf, Germany)	#MA5-15738
HAS1	1:1,200	ThermoFisher (Hennigsdorf, Germany)	#PA5-95599
HAS2	1:1,200	ThermoFisher (Hennigsdorf, Germany)	#PA5-115388
HAS3	1:1,200	ThermoFisher (Hennigsdorf, Germany)	#PA5-93099
XYLT2	1:1,000	ThermoFisher (Hennigsdorf, Germany)	#PA5-29127

### 2.8. Inhibition of ERK phosphorylation and of NF-ĸB activation in VSMCs

The activation of ERK1/2 in VSMCs was blocked by applying the inhibitor U0126 (10 µM; Cell Signalling Technology, Beverly, MA). Activation of NF-κB was blocked with the synthetic HSP-90 inhibitor 17-DMAG (15 µM; 17-dimethylaminoethylamino-17-demethoxygeldanamycin; InvivoGen, Toulouse, France) which is known to target proteins of the NF-κB family [22]. Activation of AKT was blocked using LY294002 (25 µM; Cell Signaling, Danvers, MA). The inhibitors were dissolved in DMSO and the resulting maximum DMSO concentration in the experiments did not exceed 0.05%.

### 2.9. Induction and quantification of VSMCs calcification

The calcium/phosphate (Ca/Pi)-induced calcification of VSMCs and its quantification by Alizarin red staining were performed as previously described [23].

### 2.10. Statistical analyses

Data sets were analyzed by one-way or two-way ANOVA followed by the Holm-Sidak post hoc test using GraphPad PRISM, version 6.01 (GraphPad Software, San Diego, CA), and differences with *p* values <.05 (*) were considered statistically significant.

## 3. Results

### 3.1. FGF-23 and Klotho slightly stimulate cell proliferation

**Figure 1.**
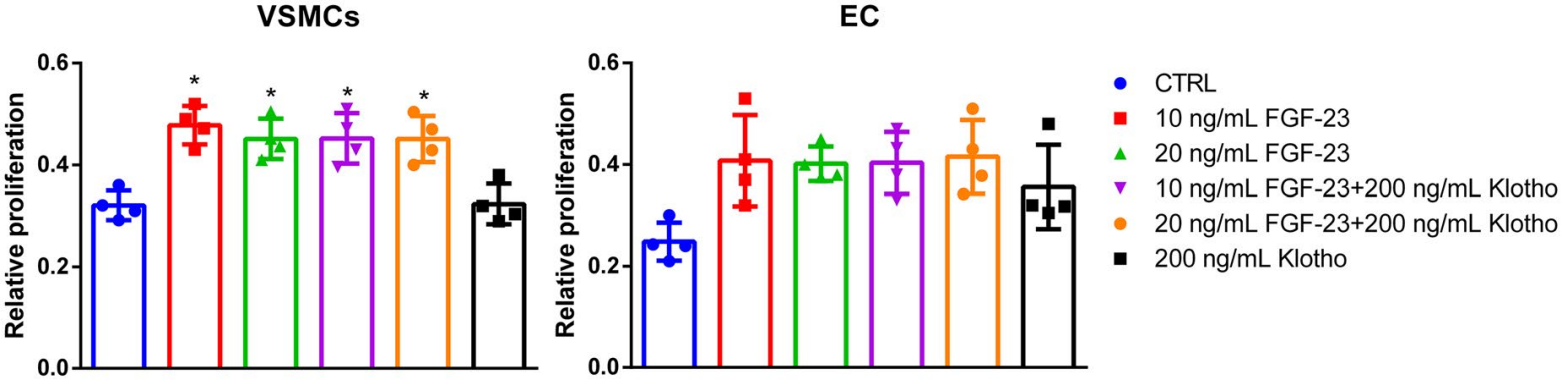
Effects of FGF-23 on the proliferation of VSMCs and ECs. Cellular proliferation of VSMCs and ECs was determined using the MTT method after treatments as indicated. Shown are means ± SD (*n* = 4; *p < .05 compared to control). ECs: endothelial cells; FGF-23: fibroblast growth factor-23; VSMCs: vascular smooth muscle cells.

An initial dose-finding experiment helped to narrow down the working concentration of FGF-23 to approximately 10–20 ng/mL (see Supplemental Fig. S1). The treatment of VSMCs and ECs with 10 and 20 ng/mL FGF-23 alone led to a ∼1.5-fold increase of proliferation compared to control. In combination with 200 ng/mL Klotho, this effect was not significantly affected (Figure 1). The treatment with 200 ng/mL Klotho alone showed no stimulatory effects in VSMCs, but an ∼1.3-fold increase of proliferation in ECs (Figure 1). However, this effect in ECs was not significant. We also analyzed if altered concentrations of FBS in the growth medium affect the effects of FGF-23 on proliferation. Supplemental Fig. S2 demonstrates that the effects of varying FGF-23 concentrations on proliferation are not markedly influenced by the FBS concentration applied. Next, we investigated whether treatment with FGF-23 also leads to a change in sGAG levels in the cells.

### 3.2. FGF-23 and Klotho increase levels of sGAGs in both cell types

**Figure 2.**
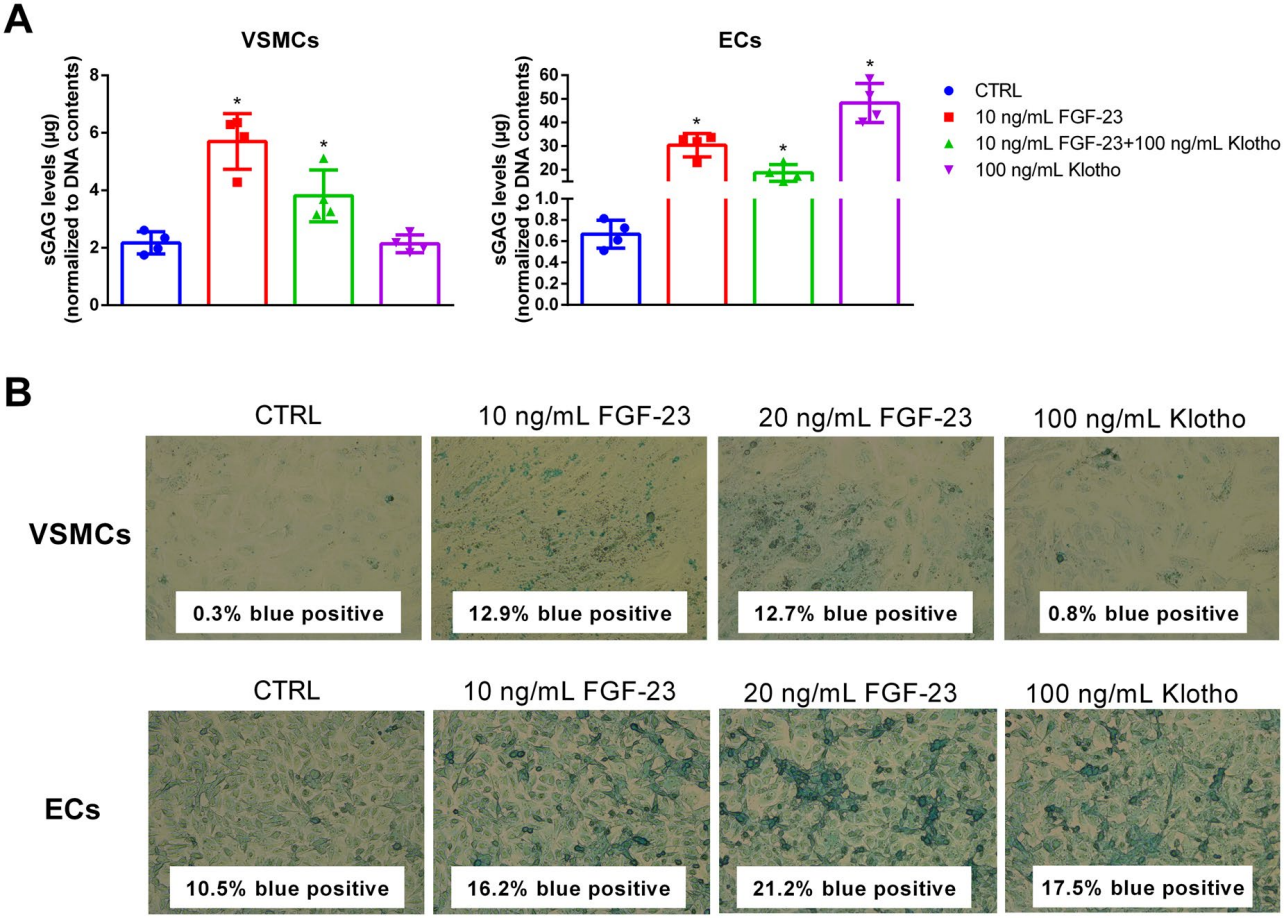
Effects of FGF-23 on levels of sGAGs in VSMCs and ECs. The cells were treated for 5 d as indicated. (A) sGAG levels of the cells were quantified using the Blyscan assay. Shown are means ± SD (*n* = 4, *p < .05 compared to control). (B) After fixation, the sGAGs in the cells were stained with Alcian blue. Digitized microscopy images were quantified using Image J (National Institutes of Health, Bethesda, MA). Shown are representative microscopy images from one out of three independent experiments. Bars = 50 µM. ECs: endothelial cells; FGF-23: fibroblast growth factor-23; sGAGs: sulfated glycosaminoglycans; VSMCs: vascular smooth muscle cells.

The Blyscan assay revealed that 10 ng/mL FGF-23 indeed significantly increased the levels of sGAGs by ∼2.5-fold in VSMCs compared to control. The combination of FGF-23 with Klotho provoked an only 1.6-fold increase of sGAGs while Klotho alone showed no effects (Figure 2(A)).

When compared to VSMCs, the effects of FGF-23 and Klotho on sGAGs in ECs were distinctly increased. The treatment with 10 ng/mL FGF-23 induced an up to ∼50-fold augmentation of sGAG levels compared to control (Figure 2(A)). The combination of FGF-23 with 200 ng/mL Klotho induced a ∼35-fold increase of sGAG levels compared to control. Interestingly, when applied without FGF-23, 200 ng/mL Klotho induced an ∼72-fold increase of sGAG levels in ECs compared to control (Figure 2(A)). In parallel, we complemented the data on effects of FGF-23 on sGAGs in the cells by Alcian blue staining. Like the Blyscan assay, we found higher sGAG levels in FGF-23 treated cells compared to control cells, and this effect was stronger in ECs than in VSMCs (Figure 2(B)). While Klotho alone did not enhance sGAG staining in VSMCs, it induced, similar to FGF-23, a markedly stronger sGAG staining in ECs (Figure 2(B)).

### 3.3. FGF-23 stimulates HA in vascular cells

**Figure 3.**
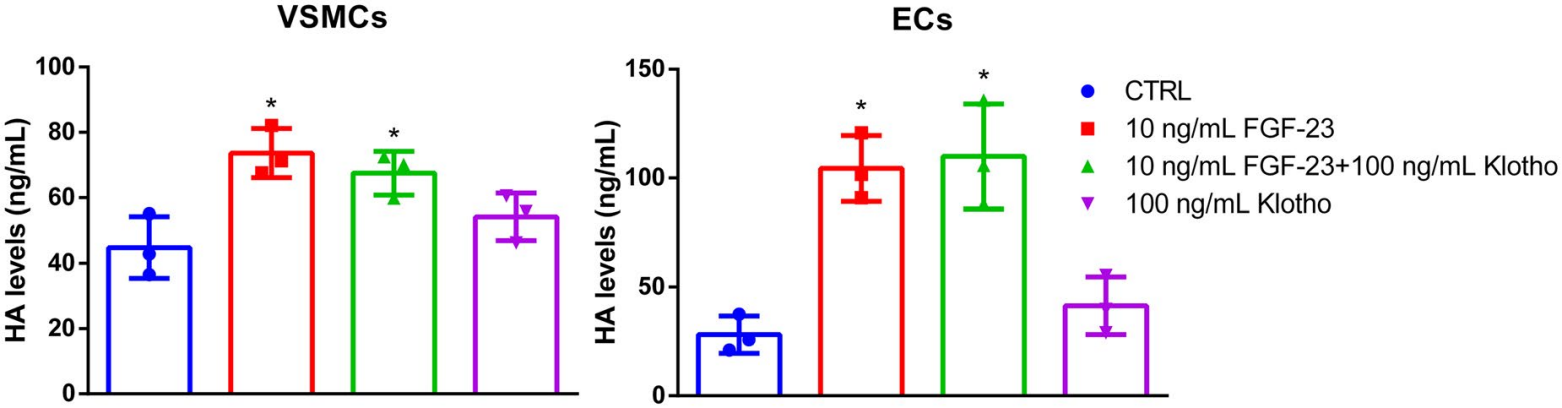
Effects of FGF-23 on HA levels in VSMCs and ECs. VSMCs and ECs were treated as indicated for 5 d. HA levels in cell lysates were determined by ELISA measurements (means ± SD; *n* = 3; *p < .05 compared to control). ECs: endothelial cells; FGF-23: fibroblast growth factor-23; HA: hyaluronic acid; VSMCs: vascular smooth muscle cells.

Besides the sGAGs, we also analyzed if FGF-23 influences the generation of the non-sGAG HA in VSMCs and ECs. FGF-23 significantly increased HA levels in VSMCs by ∼1.6-fold compared to control (Figure 3). Once again, the effects of FGF-23 were more pronounced in ECs than in VSMCs, with HA levels being increased 3.7-fold compared to the control. Further, a combination of FGF-23 with Klotho did not lead to a significant change in HA levels of both cell types. In contrast to the sGAG measurements, treatment with Klotho alone did not lead to a significant increase in HA levels (Figure 3).

### 3.4. FGF-23 induces the expression of GAG-associated genes and proteins

**Figure 4.**
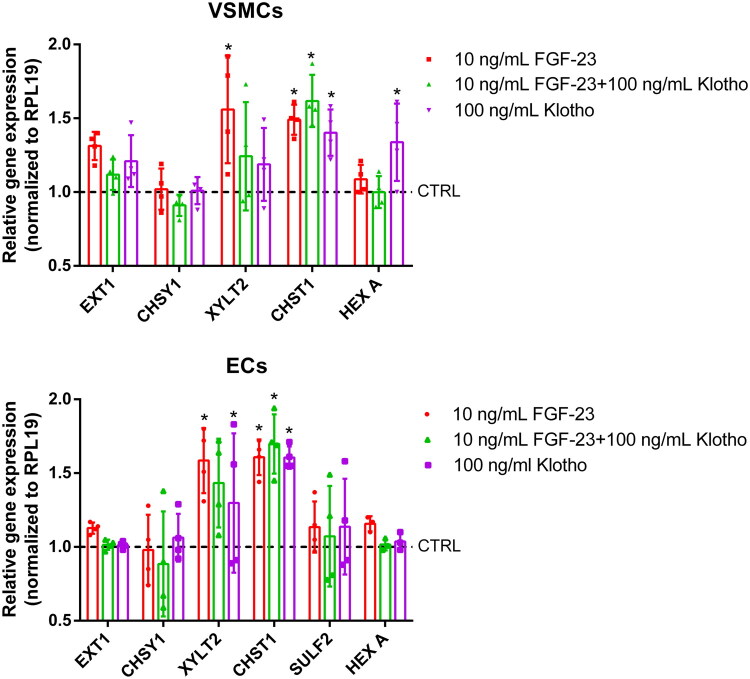
Effects of FGF-23 on GAG-relevant gene expression in VSMCs and ECs. VSMCs and ECs were treated as indicated for 24 h. Gene expressions were determined by qPCR and were normalized to RPL19 expressions. Shown are means ± SD (*n* = 4; *p < .05 compared to control). ECs: endothelial cells; FGF-23: fibroblast growth factor-23; GAGs: glycosaminoglycans; VSMCs: vascular smooth muscle cells.

We next analyzed effects of FGF-23 on the relative gene-expression of GAG-associated genes including three genes involved in GAG-synthesis (EXT1, CHSY1, and xylosyltransferase 2 (XYLT2)), one gene associated with GAG-sulfation (carbohydrate sulfotransferase 1 (CHST1)), and one with GAG-degradation (HEXA). In VSMCs, the overall effects of the different treatments were rather small. FGF-23 induced a significant ∼1.5–1.6-fold increase of XYLT2 and CHST1 gene expression (Figure 4). The effects of FGF-23 + Klotho and of Klotho alone were slightly weaker in each case, except for CHST1. Here, a combination of FGF-23 and Klotho induced an elevated gene expression of ∼1.7-fold compared to control (Figure 4).

In ECs, FGF-23 significantly upregulated the genes CHST1 and XYLT2 ∼ 1.6-fold (Figure 4(B)). The combination of FGF-23 and Klotho provoked comparable effects for both genes. Also, the treatment with Klotho alone also led to a modest yet significant upregulation of XYLT2 and CHST1 gene expression, by factors of 1.3 and 1.6, respectively. The other genes were not affected by the different treatment groups.

**Figure 5.**
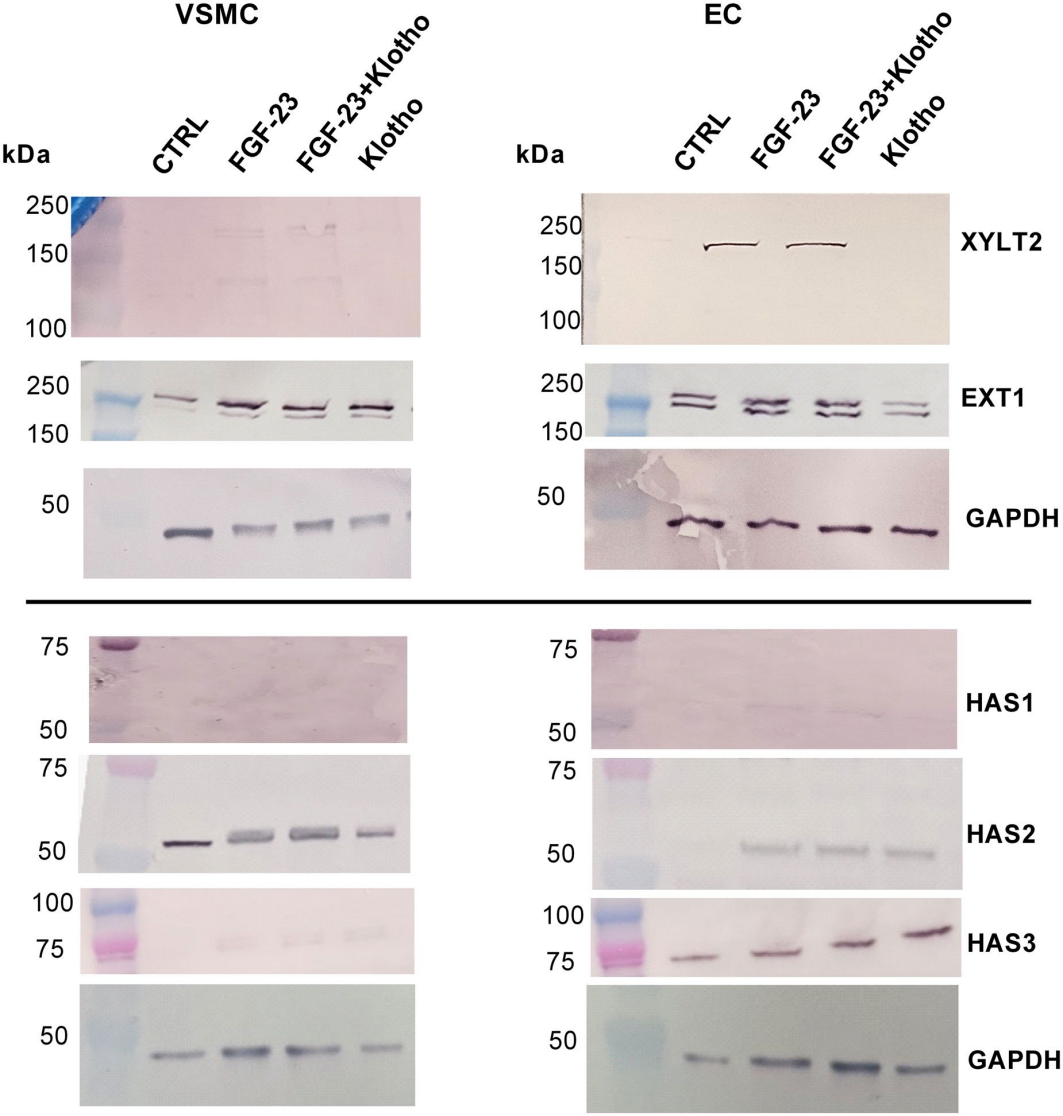
Effects of FGF-23 on GAG-relevant protein expressions in VSMCs and ECs. Cells were treated for five days with 10 ng/mL FGF-23 with or without 100 ng/mL Klotho. Protein expressions were determined by Western blot. Displayed are representative blots from one out of three independent experiments. ECs: endothelial cells; FGF-23: fibroblast growth factor-23; GAGs: glycosaminoglycans; VSMCs: vascular smooth muscle cells.

Next, we examined the protein expression of XYLT2, EXT1, and HAS1–3 as an example. Partly reflecting the gene expression analyses, the treatments with FGF-23 alone and with FGF-23 + Klotho slightly increased the protein expression of XYLT2 in VSMCs (Figure 5). Klotho alone showed no effect. Similar results were obtained from EC-derived samples (Figure 5). Both cell types also comprised higher protein expressions of EXT1 in FGF-23 and FGF-23 + Klotho treated cells (Figure 5). In VSMCs, the effects of Klotho alone were comparable to that of FGF-23, while Klotho alone induced distinctly weaker effects on EXT1 expression in ECs (Figure 5).

For the HA-synthesizing enzymes, we observed a slight stimulatory effect of FGF-23 and FGF-23 + Klotho on HAS1 expression in ECs, while in VSMCs no HAS1 was detected (Figure 5). For HAS2 protein expression, we observed a distinct increase compared to control due to all three treatments in both cell lines. In contrast, HAS3 protein expression was only slightly increased in VSMCs and ECs by all three treatments (Figure 5).

### 3.5. FGF-23 and Klotho activate FGFR-associated signaling

**Figure 6.**
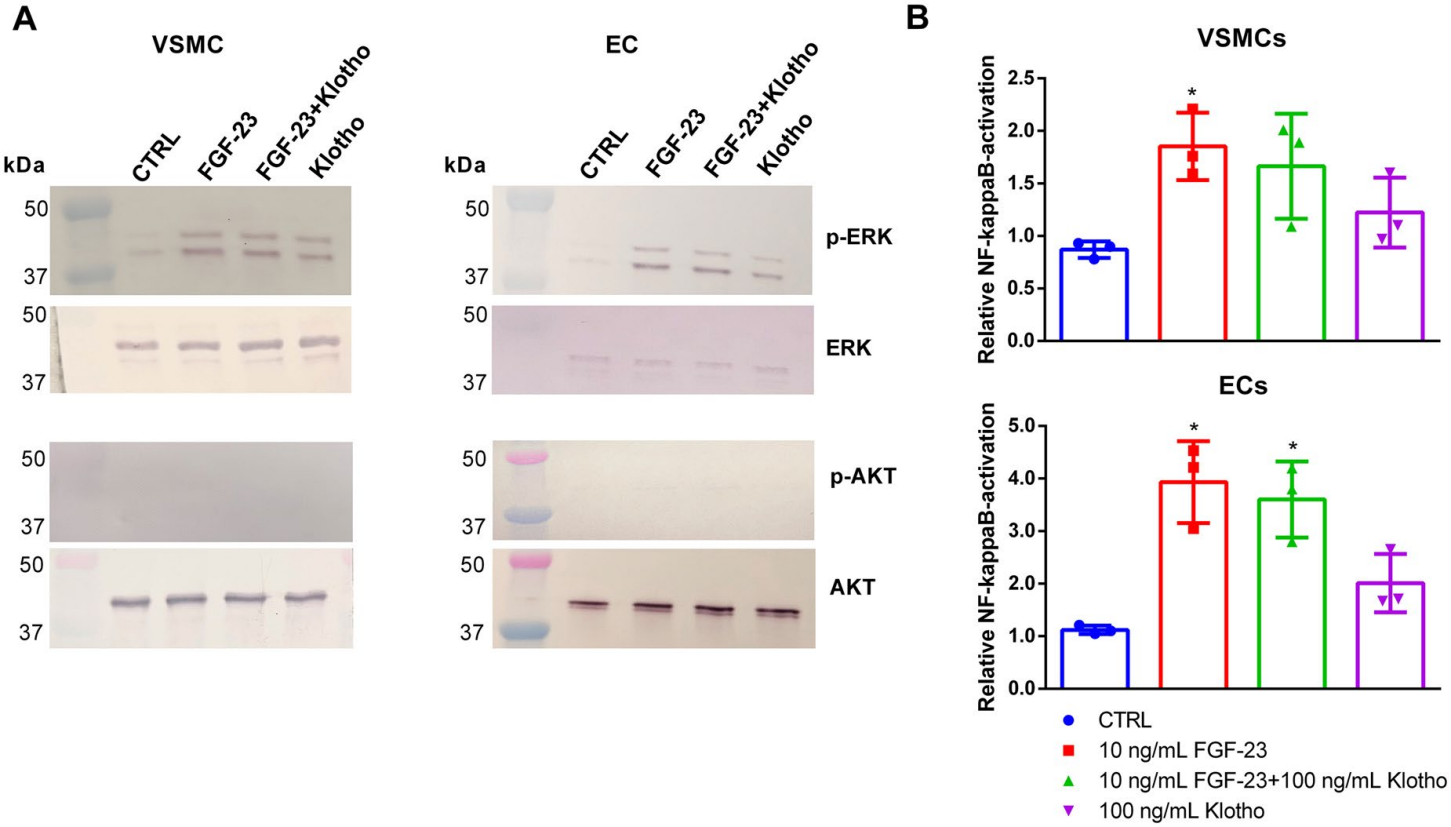
Effects of FGF-23 on activation of cellular signaling in VSMCs and ECs. (A) Cells were treated for 15 min with 10 ng/mL FGF-23 with or without 100 ng/mL Klotho as indicated. Protein expressions were determined by Western blot. Displayed are representative blots from one out of three independent experiments. (B) Cells were treated for 24 h with or without 10 ng/mL FGF-23 and/or 100 ng/mL Klotho. Activation of the transcription factor NF-κB was determined using a luminescent reporter assay (means ± SD; *n* = 3; *p < .05 compared to control). ECs: endothelial cells; FGF-23: fibroblast growth factor-23; VSMCs: vascular smooth muscle cells.

We next evaluated the underlying mechanisms of FGF-23 by studying the activation of FGFR-associated signaling pathways. Figure 6(A) shows that the treatment with FGF-23 and FGF-23 + Klotho induced a strong phosphorylation of ERK in both cell lines compared to control. Interestingly, Klotho alone also caused a slightly reduced increase in ERK phosphorylation. In contrast, no effects of the different treatment groups were observed for the phosphorylation of AKT signaling (Figure 6(A)).

FGF-23 and FGF-23 + Klotho also led to an activation of NF-κB signaling in both cell types (Figure 6(B)). The effects were significantly more pronounced in ECs than in VSMCs. In contrast to the phosphorylation of ERK, Klotho showed only minor effects on the activation of NF-κB in both cell types (Figure 6(B)).

### 3.6. Inhibition of ERK signaling and NF-κB activation alleviates the stimulating effects of FGF-23 on GAG production in ECs and VSMCs

**Figure 7.**
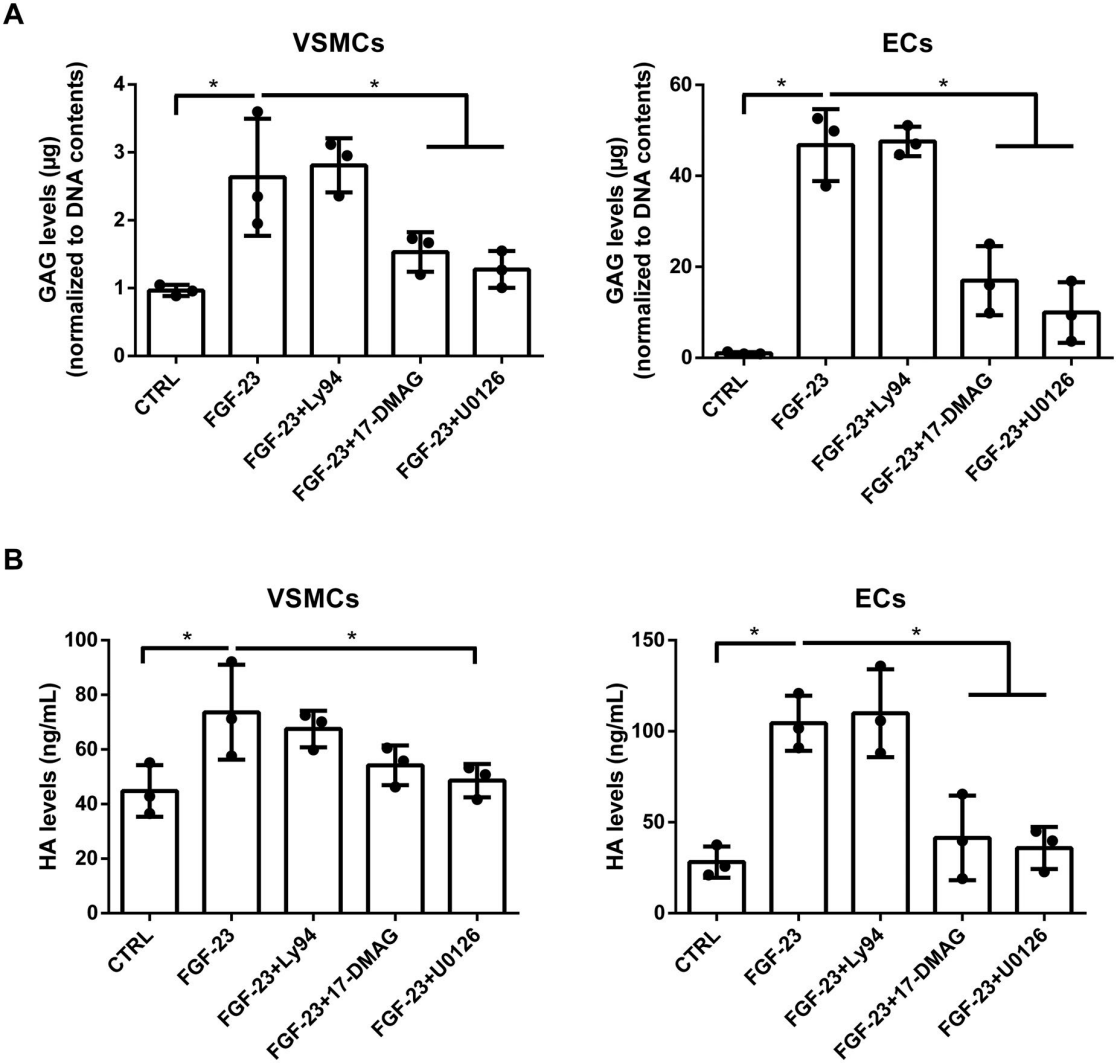
Effects of inhibition of ERK and NF-κB on FGF-23-induced GAG levels in VSMCs and ECs. The cells were treated for 5 d as indicated with or without 10 ng/mL FGF-23 and/or 100 ng/mL Klotho. To block relevant signaling pathways, the inhibitors 10 µM U0126, 15 µM 17-DMAG, or 25 µM Ly294002 were added. Levels of sGAGs and of the non-sulfated GAG HA were determined by the Blyscan assay and ELISA, respectively. Shown are means ± SD (*n* = 3; *p < .05). 17-DMAG: an inhibitor of NF-κB signaling; ECs: endothelial cells; FGF-23: fibroblast growth factor-23; GAGs: glycosaminoglycans; HA: hyaluronic acid; Ly94: Ly294002: an inhibitor of PI3K/AKT signaling; U0126: an inhibitor of ERK signaling; VSMCs: vascular smooth muscle cells.

We finally investigated if the inhibition of the above shown FGF-23-induced signaling impacts the levels of GAGs in the cells. Indeed, the presence of the MAPK-inhibitor U0126 or of the NF-κB inhibitor 17-DMAG reduced the stimulating effects of FGF-23 on the amount of sGAGs in VSMCs by ∼40% and ∼50%, respectively (Figure 7(A)). In ECs, we even observed a reduction in sGAG levels by ∼65% following NF-κB inhibition and by ∼80% following ERK inhibition (Figure 7(A)). No such effects were observed in the presence of the AKT inhibitor Ly294002 in both cell types.

In a parallel approach, we also found that U0126 and 17-DMAG blocked the effects of FGF-23 on the production of HA in ECs by ∼65% and ∼60%, respectively (Figure 7(B)). In VSMCs, only the inhibition of ERK by U0126 led to a significant attenuation of HA production by ∼35%, whereas inhibition of NF-κB resulted in a reduction of approximately 27%, which, however, was not statistically significant (Figure 7(B)).

### 3.7. Increased sGAG levels induced by FGF-23 contribute to calcium- and phosphate-induced calcification of VSMCs

**Figure 8.**
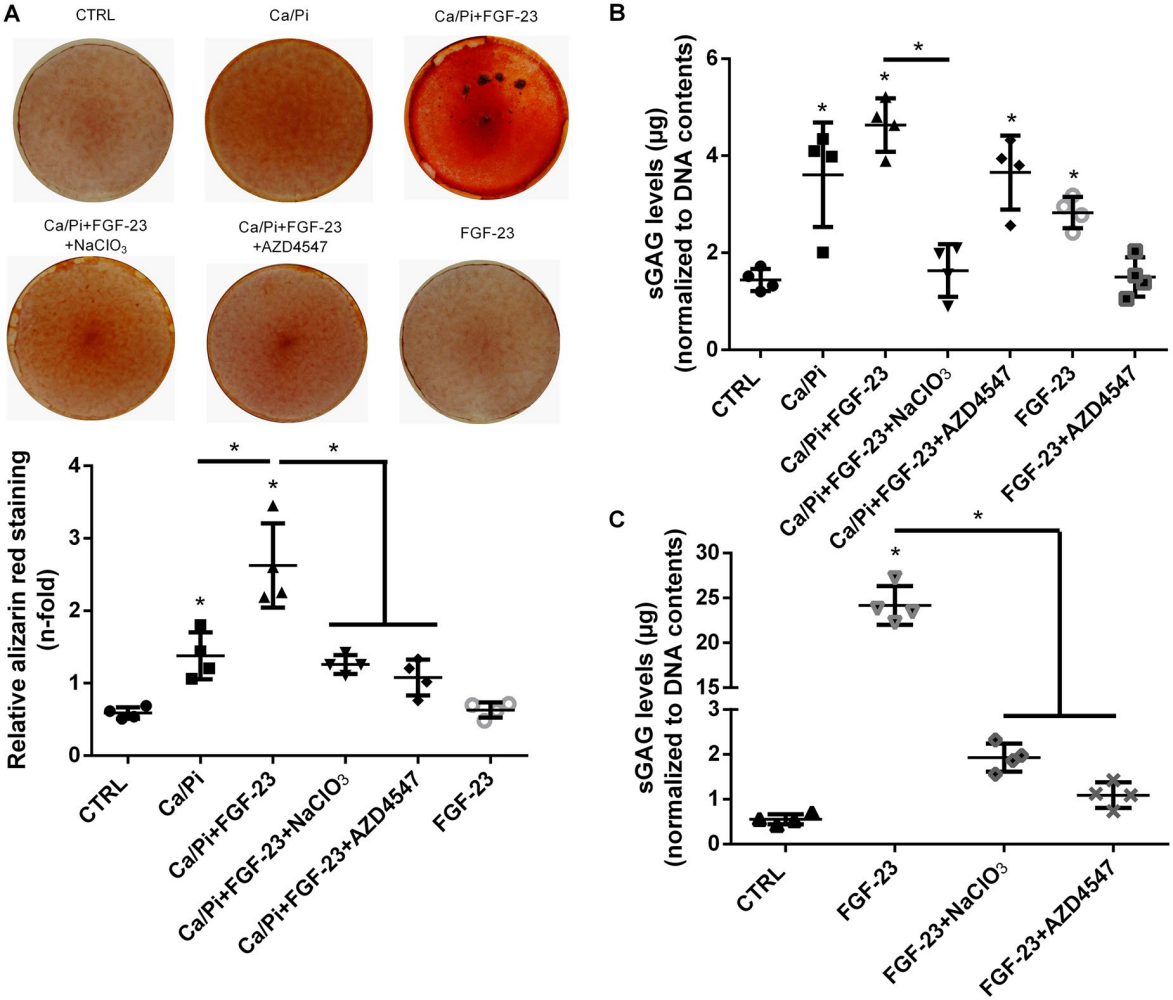
Effects of pharmacological inhibitors of GAG-sulfation and FGFR signaling on cellular calcification and FGF-23-induced GAG levels in vascular cells. (A) Calcification of VSMCs was induced by treatment with elevated concentrations of Ca/Pi with or without the presence of FGF-23, the GAG-sulfation inhibitor NaClO_3_ or the FGFR-inhibitor AZD4547. After 7 d of treatment, calcified areas were stained with alizarin red. Shown are representative images along with quantification of the dye of four independent experiments. If not indicated otherwise: *p < .05 compared to control. (B) In parallel experiments, sGAG levels of the VSMCs were quantified using the Blyscan assay. Shown are means ± SD (*n* = 4, if not indicated otherwise: *p < .05 compared to control). (C) ECs were treated for 5 d as indicated and sGAG levels of the cells were quantified using the Blyscan assay. Shown are means ± SD (*n* = 4, if not indicated otherwise: *p < .05 compared to control). AZD4547: an inhibitor of FGFRs; Ca/Pi: elevated concentrations of calcium and phosphate; FGF-23: fibroblast growth factor-23; NaClO_3_: sodium chlorate – an inhibitor of GAG sulfation.

To gain an initial impression of the biological relevance of the observed effects of FGF-23 on sGAGs, we subsequently investigated the impact of sGAGs on Ca/Pi-induced calcification of VSMCs. As expected, elevated concentrations of Ca/Pi led to a calcification of cultured VSMCs indicated by an ∼45% more intense alizarin red staining of treated cells (Figure 8(A)). The additional treatment with FGF-23 further increased the calcification of VSMCs by ∼65% (Figure 8(A)). These effects of the treatments were mirrored by the levels of sGAGs of the VSMCs (Figure 8(B)). The concomitant inhibition of sulfation of GAGs by NaClO_3_ caused a reduction in sGAG values to a level similar to that of the control group (Figure 8(B)) and effectively blocked the augmenting effects of FGF-23 on Ca/Pi-induced calcification (Figure 8(B)). Interestingly, also the presence of the pharmacological FGFR-inhibitor AZD4547 neutralized the promoting effects of FGF-23 on Ca/Pi-induced calcification and the generation of elevated sGAG levels in the cells (Figure 8(A,B)). As expected, NaClO_3_ also blocked the FGF-23-induced generation of elevated sGAG levels in ECs (Figure 8(C)). The effects of FGF-23 on sGAG levels were also abolished by FGFR inhibition using AZD4547 (Figure 8(C)).

## 4. Discussion

Aiming to better understand pathophysiological effects of FGF-23 in the cardiovascular system, the aim of the present study was to evaluate whether FGF-23 has any effects on the expression of GAGs in vascular cells. The first step was to identify a suitable working concentration for FGF-23 in our model system. In line with previous works [17], we identified 10 ng/mL as a good compromise between biological effects in VSMCs and ECs and the amount of substance to be used. The FGF-23 concentrations we use are in the ng/mL range. Typical human *in vivo* concentrations are distinctly lower and are in pg/mL range. While *in vitro* studies can only approximate the *in vivo* situation, there are also several justifications for applying higher concentrations of growth factors in cell culture experiments. The measured plasma concentration does not necessarily represent the biologically available free ligand at the cell surface: growth factors are subject to proteolytic processing, assay differences, and protein binding that affect the fraction that is active. These analytical and biological issues complicate direct comparison between a plasma pg/mL measurement and effective ligand dose at the receptor in culture [24]. Furthermore, practical aspects of *in vitro* exposure favor higher nominal doses: recombinant proteins can be lost to nonspecific adsorption to plastic or to serum proteins in the medium, and in many cell systems only a single bolus of ligand is applied (no continuous secretion). Both phenomena reduce the effective exposure over time and thus higher starting concentrations are used to maintain biologically meaningful signaling across the experimental interval. Other *in vitro* studies on FGF-23 and other growth factors report biological effects at high ng/mL concentration as well [25,26].

At 10 ng/mL, FGF-23 induced a significant increase of levels of sGAGs and of HA in both tested cell lines. To our knowledge, this effect has not been previously described in the literature. On one hand, there is some evidence suggesting that FGF-23 can influence the production of GAGs in cells by interacting with various receptors and co-factors, which can impact cellular processes, including the synthesis of GAGs [15]. On the other hand, the exact mechanisms and extent of this influence are still being studied.

FGF-23 alone had the strongest effect on both cell lines and a combination with Klotho did not lead to any increase. This in part fits to previous data where FGF-23 has been shown to affect the phenotype of VSMCs in the presence and the absence of Klotho [17]. This is often referred to as non-canonical FGF-23 signaling [27].

We also observed distinctly stronger effects of FGF-23 on GAGs in ECs compared to VSMCs and strong effects of Klotho alone in ECs. The mechanisms behind these facts are unknown. ECs generally express higher levels of Klotho compared to VSMCs, including the here used EA.Hy926 cell line [28–30]. Also, Klotho contributes significantly to the production of nitric oxide (NO) in ECs [29], while Klotho in VSMCs primarily helps to reduce oxidative stress and inflammation [28]. However, these strong effects of Klotho alone in ECs are limited to the production of sGAGs. No such effects are seen, for example, in gene expression analyses or HA levels; however, this observation is limited to the specific time point of the respective measurements, and stronger effects at other time points might be possible.

In the gene expression analyses, we observed slight effects of FGF-23 on the expression of GAG-associated genes in both cell lines. However, only gene expressions of CHST1 and XYLT2 were significantly induced after treatment for 7 d with FGF-23. CHST1 belongs to the keratan sulfotransferase family which influences the structure and the degree of sulfation of cellular GAGs [31]. XYLT2 encodes for a xylosyltransferase that initiates the biosynthesis of GAG chains of chondroitin sulfate, heparan sulfate, heparin, and dermatan sulfate [32]. Our data from the sGAG assay and the Alcian blue staining indicate that increased expression levels of both genes are likely to contribute to more synthesis of sGAGs in both cell types.

Our results from gene and protein expressions revealed stimulatory effects of FGF-23 on the remodeling of GAGs. A study by Li et al. [33] found a high endogenous expression of CHST genes in the EA.Hy926 cell line that we also used in our study. Besides an increased sulfation of GAGs, the effects of an additional stimulation of CHST1 expression by FGF-23 might also explain the stronger effects on sGAG levels in ECs compared to VSMCs. However, the markedly stronger effect of Klotho on sGAG levels compared to FGF-23 cannot be explained by the regulation of CHST1 alone. This suggests the involvement of other sulfotransferases or sulfate transporters, which will be the focus of future Klotho-specific follow-up studies.

Xylosyltransferases are essential enzymes in catalyzing the initial step in the assembly of GAG chains on core proteins [32]. Their dysregulation, such as the observed upregulation of XYLT2 by FGF-23, can lead to alterations in cell–cell interactions, inflammation, and vascular remodeling, contributing to conditions such as atherosclerosis and heart failure [34].

Regarding the rather moderate effects of FGF-23 on the expression of GAG-related genes shown here, it should be noted that these data reflect gene expression at a single, predefined time point − 24 h after treatment. While the results do indicate regulatory effects of FGF-23, accurate quantification at the transcriptional level would require detailed kinetic studies for each individual gene.

Besides the effects of FGF-23 on sGAGs, we also observed a stimulating effect of FGF-23 on HA levels. This fact is consistent with the observed upregulation of hyaluronan synthase (HAS) proteins in both cell lines due to the treatment with FGF-23. HAS catalyzes the formation of HA chains by linking alternating glucuronic acid and N-acetylglucosamine residues via β-1,3 and β-1,4 glycosidic bonds [35]. HAS2 affects HA production in many cell types including VSMCs [36,37], while HAS3 synthesizes shorter HA chains, mainly during inflammation [38].

Increased levels of HA can promote the proliferation and migration of ECs and VSMCs [39,40]. In addition, it contributes to the progression and increased disruption of vascular integrity in atherosclerosis [41] and can influence proinflammatory processes such as the recruitment of macrophages [42].

In addition to the effects of FGF-23 on HA levels observed here, other growth factors and cytokines such as the platelet-derived growth factor or transforming growth factor-beta are known to increase HAS expression and, subsequently, HA synthesis [43,44]. We recently also described HA-stimulating effects of the inorganic UT inorganic phosphate and of the organic UTs indoxyl sulfate and p-cresyl sulfate in ECs and VSMCs [6]. Since FGF-23 is considered to also be a UT [14,21], the effects of FGF-23 fit the preceding observations.

Aiming to gain insight into the mode of action of FGF-23, we found that FGF-23 induces the activation of the signaling molecule ERK and of NF-κB signaling in both cell lines. Indeed, the inhibition of both pathways significantly attenuated the effects of FGF-23 on GAG levels in ECs and VSMCs. The known activation of the ERK signaling pathway as a part of the broader MAPK pathway by FGF-23 plays a crucial role in cell proliferation, differentiation, and survival [45]. For instance, the activation of this FGF-23-ERK/MAPK axis can lead to increased inflammation, oxidative stress, and cell apoptosis in conditions such as acute kidney injury [45]. Further, ERK signaling also impacts the effects of FGF-23 in regulating phosphate and vitamin D metabolism [46]. For example, renal phosphate reabsorption is inhibited by FGF-23-mediated ERK activation [47].

An interaction of FGF-23 with NF-κB has also been described before, e.g., in the context of inflammatory responses [18]. For instance, during kidney disease, FGF-23 can induce inflammation and fibrosis through the activation of the NF-κB pathway [48]. This involves the interaction of FGF-23 with specific receptors and co-receptors, such as FGFR and α-Klotho, which then trigger respective downstream signaling cascades [48]. Consistent with previous findings, our study suggests that FGFRs may serve as target receptors for FGF-23 in the vascular cells used here, as pharmacological inhibition of FGFR abolished the FGF-23-induced effects on sGAG levels.

The molecular links between CKD and systemic and vascular inflammation and oxidative stress is incompletely understood [20]. FGF-23 stimulates NF-κB, which is considered a key mediator of vascular inflammation [49] and the subsequently upregulated sGAGs are also linked to inflammation, e.g., via their interaction with chemokines [12]. Thus, the GAG-specific effects of FGF-23 and of other UTs [6,7,21] could be an expression of a generalized inflammation and may represent a building block to explain the onset and progression of inflammation in CKD. Of course, the latter requires a targeted study in the animal model.

Our study has some obvious limitations. This study offers only a preliminary view of FGF-23- or Klotho-specific GAG regulation, including sulfation. Future research should involve broader gene/protein expression profiling including kinetics and functional analyses. Further, the *in vitro* static cell culture conditions are not representative of physiological conditions where vascular cells are consistently exposed to laminar blood flow and changing concentrations of soluble blood components (e.g., of UTs). This could have an impact on the cell surface such as the structure of the glycocalyx [50]. Further, human recombinant FGF-23 and Klotho were used to treat both human ECs and rat VSMCs. However, studies show that human FGF-23 influences the cellular behavior of rodent cells. For instance, human FGF-23 has been shown to activate ERK1/2 signaling in murine VSMCs thereby affecting VSMC calcification [51]. Further, human FGF23 was shown to alleviate hypophosphatemia in mice [52] and also to influence high phosphate-induced vascular calcification in rat VSMCs [53]. Finally, as outlined above, the FGF-23 concentrations applied were considerably higher than plasma levels observed *in vivo*, a fact that should be taken into account when interpreting the effects. A final point is the usage of U0126 as a MEK/ERK pathway inhibitor. Data should be interpreted with caution, as U0126 may also affect voltage-gated potassium channels. U0126 has been reported to modulate Kv1.5 expression in VSMCs under hypoxia [54] and to directly block several Kv subtypes independently of MEK inhibition [55]. Given that both VSMCs, including A7r5 [56], and ECs [57,58] express functional Kv channels, potential off-target effects cannot be excluded.

Regarding the probable *in vivo* relevance of our findings, several studies have shown that changes in GAG metabolism impact vascular complications associated with CKD. For instance, dialysis patients, who exhibit markedly elevated plasma concentrations of FGF-23, also show pronounced degradation of the endothelial glycocalyx [59]. This leads to increased circulating levels of the GAGs heparan sulfate and HA and is associated with endothelial dysfunction and vascular leakage [59]. Such a degradation of the endothelial glycocalyx also correlates with elevated UT levels and reduced vascular reactivity in CKD patients [60]. In recent years, GAGs themselves have increasingly come into focus in vascular research. Highly sGAG chains are responsible for an enhanced binding of low-density lipoproteins (LDLs) in vessel walls which contributes to atherogenesis [61] and promotes foam cell formation due to an altered oxidative modification of LDLs [62]. High levels of GAGs also contribute to vascular calcification by promoting osteogenic differentiation of VSMCs [11,63]. Vice versa, this also enables sGAGs as therapeutic targets. An antibody targeting sGAGs reduced atherosclerotic lesion formation in rabbit and mouse models [64] which also strengthens the causal role of sGAGs in vascular disease. This last point is also supported by our current data. Inhibition of sGAG sulfation by NaClO_3_, an inhibitor of the sulfate donor 3′-phosphoadenosine 5′-phosphosulfate (PAPS) [65], led to a reduction in Ca/Pi-induced calcification of VSMCs. Thus, this study provides the first evidence that FGF-23 directly affects vascular extracellular matrix composition and promotes calcification, beyond its indirect effects on phosphate homeostasis, although *in vivo* confirmation is still required. Since increased FGF-23 levels are widely accepted as an emerging factor in CKD–mineral and bone disorder (CKD–MBD) [66,67], the present study, in addition to previous findings [68], provides further evidence that FGF-23 is actively involved in the pathophysiology of vascular calcification.

**Figure 9.**
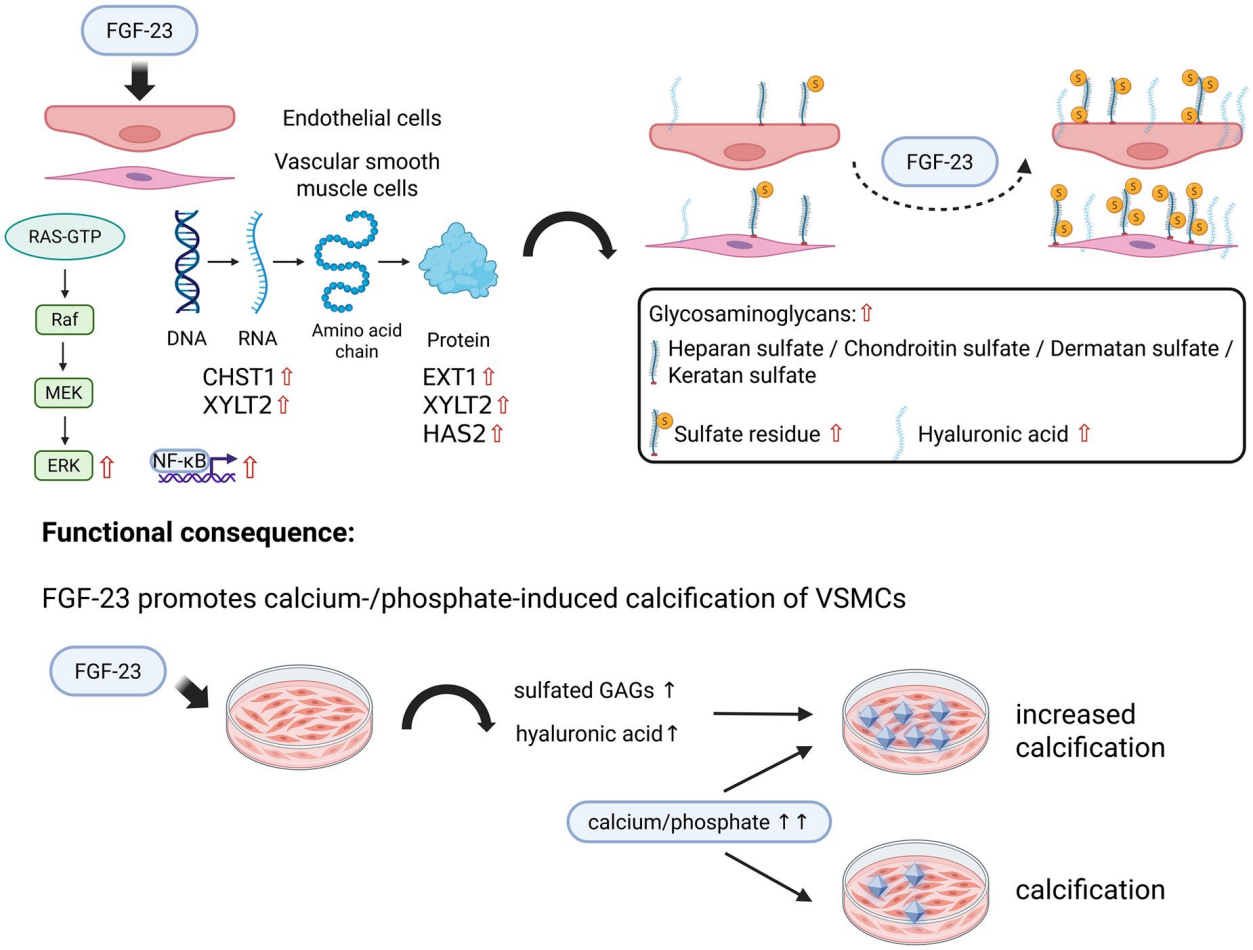
Schematic summary of the effects of FGF-23 observed in vascular cells in this study. FGF-23 induces activation of ERK and NF-κB in ECs and VSMCs which is accompanied by increased expression of genes and proteins being involved in the biosynthesis and sulfation of GAGs. This finally leads to increased levels of sulfated GAGs and of hyaluronic acid in the cells. Functionally, the elevation of sulfated GAG levels induced by FGF-23 promotes calcium- and phosphate-driven calcification of VSMCs. Created in BioRender. Hahndorf, J. (2025) https://BioRender.com/zkdqg2v.

Figure 9 provides a schematic illustration of the effects of FGF-23 in vascular cells as shown and discussed in this study.

Our data, together with the additional findings outlined here, suggest some directions for translating these findings into clinical research. Circulating or tissue GAG-profiles might be used as biomarkers for early pathophysiological vascular damage in CKD. This demands well-characterized CKD patient cohorts and correlation with vascular outcomes. GAG-profiles may also represent a target for pharmacological interventions – such as phosphate binders or Klotho modulators – with the potential to attenuate vascular calcification in CKD models or selected patient subgroups. Additional approaches for therapeutic intervention may involve modulation of the FGF-23/GAG axis described here. Such approaches – e.g., by using FGFR inhibitors, sulfotransferase inhibitors, or by influencing Klotho activity – could provide novel therapeutic strategies for CKD-associated vascular pathologies. However, these concepts will require further dedicated investigation.

In conclusion, FGF-23 increases levels of sGAGs and HA in vascular cells via activation of ERK and NF-κB-signaling with implications for vascular calcification. This GAG-specific remodeling of the extracellular matrix might contribute to the FGF-23 mediated further development of CVD during CKD.

## Supplementary Material

Supplemental figures.docx

## Data Availability

The data that support the findings of this study are available from the corresponding author upon reasonable request.
