# Supplementary material for: Fibroblast growth factor-23 remodels vascular extracellular matrix via glycosaminoglycan induction: implications for calcification in chronic kidney disease
Source: Ren Fail. 2025 Oct 6;47(1):2567528. doi: 10.1080/0886022X.2025.2567528 (PMC12507111; doi:10.1080/0886022X.2025.2567528)
Supplement: Supplemental figures.docx [file IRNF_A_2567528_SM3911.docx]

**Supplemental content to…**

**“Fibroblast Growth Factor-23 Remodels Vascular Extracellular Matrix via Glycosaminoglycan Induction: Implications for Calcification in Chronic Kidney Disease”**

Christian Freise^1^, Tia Jernej^1^, Susanne Metzkow^1^, Jörg Schnorr^1^ and Matthias Taupitz^1^

^1^Charité - Universitätsmedizin Berlin, corporate member of Freie Universität Berlin and Humboldt-Universität zu Berlin, Department of Radiology, Charitéplatz 1, 10117 Berlin, Germany

**Supplemental Figure S1**

**

**

**Fig. S1. Effects of different FGF-23 concentrations on the proliferation of VSMCs and ECs.** The proliferations of VSMCs and ECs were determined using the MTT method after FGF-23 treatments as indicated in DMEM containing 10% FBS. Shown are individual replicates with means connected. *Abbr.: ECs, endothelial cells; FBS, fetal bovine serum; FGF-23, fibroblast growth factor-23; VSMCs, vascular smooth muscle cells.*

**Supplemental Figure S2**

**

**

**Fig. S2. Effects of FGF-23 on the proliferation of VSMCs and ECs in serum-reduced conditions.** The proliferations of VSMCs and ECs were determined using the MTT method after FGF-23 treatments as indicated in DMEM containing 1% FBS. Shown are individual replicates with means ± SD. *Abbr.: ECs, endothelial cells; FBS, fetal bovine serum; FGF-23, fibroblast growth factor-23; VSMCs, vascular smooth muscle cells.*
